# NumMolFormer: an explicit functional group number-guided framework for structure-based drug design

**DOI:** 10.1093/bioinformatics/btag164

**Published:** 2026-04-07

**Authors:** Zhicun Zeng, Yifan Wu, Zhangli Lu, Min Li

**Affiliations:** School of Computer Science and Engineering, Central South University, Changsha, Hunan 410083, China; School of Computer Science and Engineering, Central South University, Changsha, Hunan 410083, China; School of Computer Science and Engineering, Central South University, Changsha, Hunan 410083, China; School of Computer Science and Engineering, Central South University, Changsha, Hunan 410083, China

## Abstract

**Motivation:**

Rational molecule generation that balances binding affinity with favorable physicochemical properties remains a formidable challenge in structure-based drug design. The number of functional groups is a key determinant, as over-functionalization compromises physicochemical properties, whereas under-functionalization reduces binding affinity. However, current methods are limited in their capacity to incorporate the constraint.

**Results:**

To address this, we present NumMolFormer, a Transformer-based framework designed to explicitly model functional group numbers. NumMolFormer adopts a dual-sequence input strategy, integrated with a numerical embedding module and a dual-stream differential attention mechanism, allowing molecular structures and functional group numbers to be encoded separately. This formulation alleviates the inherent limitations of standard Transformer in handling numerical information. In addition, we construct a large-scale dataset of 18 million molecules with functional group annotations for molecular pre-training, and further fine-tune the model using a combination of self-supervised learning and reinforcement learning under protein pocket constraints. The results demonstrate that NumMolFormer effectively leverages functional group information to generate molecules with improved binding affinity, synthetic accessibility, and drug-likeness compared to baseline methods.

**Availability and implementation:**

The source code and datasets are available at http://www.github.com/zengzhicun/nummolformer.

## 1 Introduction

Structure-based drug design (SBDD) aims to identify high-quality molecules that bind to protein targets by leveraging structural information (Ferreira et al. 2015, Van Montfort and Workman 2017). Conventional SBDD relies on virtual screening from existing chemical libraries, which cover only a tiny fraction of the drug-like chemical space, approximately $10^{8}$ molecules out of a theoretically estimated $10^{60}$, leaving many potentially active compounds unexplored (Irwin and Shoichet 2005, Corsello et al. 2017). To efficiently explore this vast chemical space, recent advances in deep learning are pivoting SBDD toward generative approaches, enabling the direct design of novel ligands.

Early deep learning-based generative methods typically follow a *bottom-up* strategy, constructing ligands at the atomic level. Representative approaches include geometric models, which represent protein–ligand complexes as 3D atom-level graphs or point clouds (Liu et al. 2022b, Guan et al. 2023b), as well as sequential models that generate molecules as tokenized SMILES strings (Zhou et al. 2023, Kong et al. 2024). While effective at modeling local interactions, such atom-centric formulations often lacks global planning over functional substructures, leading to limited synthetic feasibility.

Consequently, research has increasingly adopted *top-down* strategies that utilize functional groups or pharmacophores as fundamental units, thereby directly incorporating medicinal chemistry knowledge. Recent works, such as PGMG (Zhu et al. 2023) and TransPharmer (Xie et al. 2025), leverage functional group spatial information or fingerprints to guide generation. By applying qualitative constraints on functional groups, these approaches markedly enhance the drug-likeness of the generated molecules.

Despite their utility, current functional group-guided approaches are hindered by a critical oversight: while they identify necessary chemical moieties, they fail to quantitatively constrain the number of each functional group during molecular design. The lack of precise structural control undermines the druggability of generated candidates (Mao et al. 2016, Ertl et al. 2020). Indeed, based on the principle of Ligand Efficiency, over-functionalization often increases synthetic complexity and leads to suboptimal binding geometries, whereas insufficient substitution typically results in diminished binding affinity (Hann et al. 2001, Li 2020). Moreover, such precision is vital given the prevalence of Activity Cliffs in medicinal chemistry, where minute structural modifications can trigger disproportionate collapses in biological activity (Lin and Lu 1997).

Methodologically, although the Transformer architecture has established itself as the de facto standard for generative tasks, it suffers from inherent limitations in inductive bias regarding continuous numerical values and precise counting (Achiam et al. 2023, Dziri et al. 2023). Standard Transformer typically discretizes continuous values into separate tokens. Such tokenization disrupts the inherent ordinality and metric relationships within numerical data, consequently impairing the model’s capacity for numerical reasoning—including tasks like magnitude comparison and arithmetic operations (Thawani et al. 2021).

**Figure 1:**
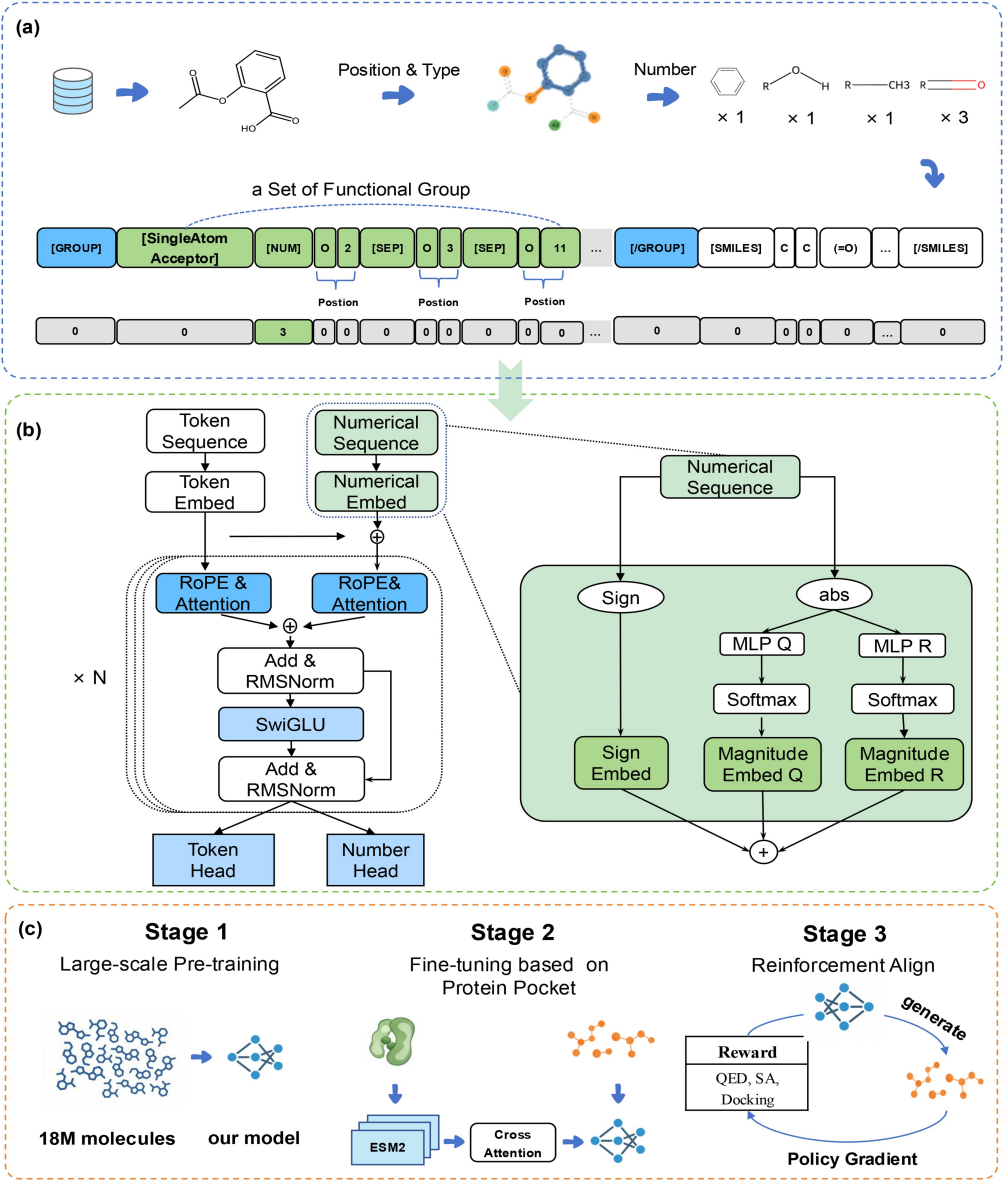
Overview of the NumMolFormer framework. (a) Construction of text-numerical dual-sequences, mapping extracted functional group features into aligned textual and numerical sequences. (b) Model Architecture. The framework presents a novel Transformer-based architecture built upon dual-stream differential attention and an Enhanced Numerical Embedding Module (inset). The latter explicitly captures numerical semantics by decomposing scalars into sign and magnitude components via MLP-based bucketization. (c) Three-stage training pipeline consisting of unconditional molecular pre-training, pocket-conditional self-supervised learning, and reinforcement learning.

To address the challenges, we propose NumMolFormer, an explicit functional group number-guided framework for structure-based drug design. Our approach explicitly represents functional groups as fine-grained triplets (*Type, Number, Position*), illustrated in Fig. 1a. This representation explicitly encodes the quantitative and spatial features required for rational drug design.

We next redesign the Transformer architecture to mitigate its limitations in numerical reasoning as shown in Fig. 1b by introducing a dual-sequence input strategy, which comprises numerical sequences and chemical text sequences, a numerical embedding module, and a dual-stream differential attention mechanism. This architecture explicitly injects functional group numbers information and decouples the quantitative data from chemical textual semantics, thereby significantly enhancing the numerical presentation.

To deal with the scarcity of high-quality protein–ligand complex data, we adopt three-stage progressive training strategy as shown in Fig. 1c. In the first stage, unconditional pre-training is performed on a large-scale dataset of 18 million molecules to learn fundamental chemical structures. In the second stage, fine-tuning is conducted by incorporating pocket information through cross-attention, using protein features encoded by the protein language model “Evolutionary Scale Modeling” (ESM2) (Lin et al. 2022). In the third stage, we employ reinforcement learning (RL) alignment with Policy Gradient, optimizing the model via a multi-objective reward function.

Our main contributions are summarized as follows:

1. *Quantitative functional group control in SBDD.* NumMolFormer utilizes the number of functional groups as prior information in drug design based on protein pocket, demonstrating its ability to generate molecules with high biological activity, synthetic accessibility and druggability.
2. *Architectural innovation for numerical reasoning.* We propose a dual-sequence architecture to separately encode molecular structure and functional-group quantities, along with an explicit numerical module and a dual-stream differential attention mechanism. This design mitigates the standard Transformer’s inductive bias toward continuous values, allowing scalar constraints to be decoupled from chemical semantics.
3. *Large-scale quantitatively annotated functional group dataset.* We construct a large-scale pre-training dataset of 18 million examples annotated with fine-grained *(Type, Number, Position)* triplets, providing the model with explicit quantitative prior knowledge to guide the generation process.

## 2 Materials and methods

### 2.1 Functional group identification

All molecules were converted to canonical SMILES using RDKit (Landrum et al. 2013) for unambiguous representation. Furthermore, functional group features were extracted using RDKit’s base feature definitions (https://github.com/rdkit/rdkit/blob/master/Data/BaseFeatures.fdef) to integrate chemical domain knowledge, as shown in Fig. 1a.

To ensure data consistency, absent functional groups were assigned a count of zero, with the placeholder [NONE] retaining their positional information. This padding strategy ensured strict alignment across all samples and enabled efficient batch training.

Formally, for a given molecule *M*, its set of functional groups is represented as


(1) $$F={\left\{(c_{i},n_{i},p_{i})\right\}}_{i=1}^{T}$$

where $c_{i}$ denotes the type of the *i*-th functional group, $n_{i}$ is its corresponding number, and $p_{i}$ provides a structured representation of its positions within the molecule.

Specifically, $p_{i}$ can be expanded as follows:


(2) $$\begin{array}{c} p_{i}=\{P_{i}^{\left(1\right)},\ldots ,P_{i}^{\left(k_{i}\right)}\}\\ P_{i}^{\left(j\right)}=\{a_{1}^{\left(j\right)},\ldots ,a_{m_{j}}^{\left(j\right)}\} \end{array}$$

Here, $k_{i}$ denotes the frequency of functional group $c_{i}$ within the molecular graph. Each instance $P_{i}^{\left(j\right)}$ is defined by a set of $m_{j}$ constituent atoms, where $a_{l}^{\left(j\right)}$ specifies the elemental type and unique positional index of each atom.

### 2.2 Dual-sequence input strategy

We introduce a text-numerical dual-sequence (Fig. 1a) to separate discrete textual semantics from functional group numbers, constructing an enriched molecular representation based on extracted features. Specifically, a textual sequence is derived from predefined templates to structurally encode functional group types, site indices, and semantic context while preserving the original SMILES notation. Concurrently, a numerical sequence provides strictly aligned numbers corresponding to the textual components.

Furthermore, we introduce two special tokens, [NUM] and [SEP], to differentiate between multiple instances of the same functional group.

### 2.3 Model architecture

NumMolFormer constructs a novel numerical embedding module and a dual-stream differential attention mechanism to model numerical semantic information. The overall architecture is illustrated in Fig. 1b.

#### 2.3.1 Numerical embedding

To better capture functional group numbers as structured signals, we design an enhanced numerical embedding module that decomposes each scalar value $n_{i}$ into three complementary components: (i) a raw numerical injection to preserve the original scale, (ii) a discrete sign embedding to represent directionality, and (iii) a soft magnitude quantization embedding to encode continuous scales.

*Raw numerical injection.* To retain the raw numerical information, the scalar value $n_{i}$ is broadcast and added to the token embedding $e_{i}^{\mathrm{text}}$:


(3) $$e_{i}^{\text{raw}}=e_{i}^{\mathrm{text}}+n_{i}$$

where $w_{i}$ denotes the standard token embedding.

*Sign embedding.* We further encode the sign of $n_{i}$ as a discrete categorical feature:


(4) $$s_{i}=\mathrm{sign}(n_{i})\in \{-1,0,+1\}\;e_{i}^{\text{sign}}={\;\text{Embed}}_{\text{sign}}(s_{i})$$

where ${\text{Embed}}_{\text{sign}}$ is a learned embedding layer that distinguishes positive, negative, and zero values.

*Soft magnitude quantization embedding.* The absolute magnitude $\left|n_{i}\right|$ is projected through two parallel lightweight feedforward networks and softly assigned to *K* learnable bins:


(5) $$p_{i}^{\left(q\right)}=\mathrm{softmax}(W_{2}^{\left(q\right)}\sigma (W_{1}^{\left(q\right)}|n_{i}|+b_{1}^{\left(q\right)})+b_{2}^{\left(q\right)})$$

(6) $$p_{i}^{\left(r\right)}=\mathrm{softmax}(W_{2}^{\left(r\right)}\sigma (W_{1}^{\left(r\right)}|n_{i}|+b_{1}^{\left(r\right)})+b_{2}^{\left(r\right)})$$

where σ is the GELU activation. Let ${\left\{v_{k}^{\left(q\right)}\right\}}_{k=1}^{K}$ and ${\left\{v_{k}^{\left(r\right)}\right\}}_{k=1}^{K}$ denote two magnitude embedding tables. The final magnitude embedding is given by:


(7) $$e_{i}^{\text{mag}}=\sum_{k=1}^{K}p_{ik}^{\left(q\right)}v_{k}^{\left(q\right)}+\lambda \sum_{k=1}^{K}p_{ik}^{\left(r\right)}v_{k}^{\left(r\right)}$$

where λ is a learnable scaling parameter.

*Final numerical embedding.* The overall numerical embedding is the combination of the three channels:


(8) $$e_{i}^{\text{num}}=e_{i}^{\text{raw}}+e_{i}^{\text{sign}}+e_{i}^{\text{mag}}$$

Numerical embedding offers three systematic advantages: (i) direct embedding of raw values to preserve *precision*; (ii) *continuity* to capture scalar variations; and (iii) explicit modeling of *numerical structure* via Sign and Soft Magnitude Quantization embeddings.

#### 2.3.2. Dual-stream differential attention

We further introduce a dual-stream differential attention mechanism to disentangle text and numerical contributions. Let $E_{\text{text}}\in R^{L\times d}$ denote the text embedding and $E_{\text{fusion}}=E_{\text{text}}+E_{\text{num}}$ represent the integrated numerical-text embedding. The self-attention mechanism is then applied in parallel across the two streams:


(9) $$H^{\text{text}}=\text{Attention}(E^{\text{text}},E^{\text{text}},E^{\text{text}\;})$$

(10) $$H^{\text{fusion}}=\text{Attention}(E^{\text{fusion}},E^{\text{fusion}},E^{\text{fusion}})$$

The incremental effect of numerical features is then isolated via subtraction:


(11) $$H^{\text{diff}}=H^{\text{fusion}}-H^{\text{text}}$$

Thereafter, the differential signal is integrated with the fused embeddings through a residual connection and normalization.


(12) $$E^{\prime }=\mathrm{Norm}(E^{\text{fusion}}+H^{\text{diff}})$$

followed by a SwiGLU and normalization transformation.

### 2.4. Training strategy

At present, high-quality experimental data for protein-ligand complexes are notably limited. The PDBbind dataset (Liu et al. 2017), currently the most extensive resource, comprises fewer than 20 000 complexes. Even with the inclusion of augmented datasets generated via docking simulations, such as CrossDocked 2020 (Francoeur et al. 2020), the total volume remains around 100 000 samples—a scale insufficient for training robust machine learning models from scratch. To circumvent this data scarcity, we employ a pre-training and fine-tuning strategy.

#### 2.4.1. Unconditional molecular pretraining

The objective of this stage is not to generate molecules with target-specific bioactivity, but to capture the implicit molecular syntax. Through this approach, we embed the chemical knowledge from vast unlabeled datasets into the model.

To enable our dual-channel autoregressive model to capture both functional group and molecular sequence information, as well as the functional group numbers, we employ a composite loss function. The loss jointly predicts the next textual token in the sequence and the numerical value representing functional group numbers. Formally, let ${\hat{s}}_{\text{tok}}$ denote the predicted token sequence, $s_{\text{tok}}$ the ground-truth token sequence, ${\hat{s}}_{\text{num}}$ the predicted numerical sequence, and $s_{\text{num}}$ the ground-truth numerical sequence. The loss is defined as:


(13) $$L(\hat{s},s)=\text{CE}({\hat{s}}_{\text{tok}\;},s_{\text{tok}})+\text{MSE}({\hat{s}}_{\text{num}},s_{\text{num}})$$

where CE(·) denotes the cross-entropy loss and MSE(·) denotes the mean squared error loss.

#### 2.4.2. Self-supervised fine-tuning on protein pocket conditions

The pretraining stage enables the model to acquire a semantic understanding of fundamental chemical principles, but it lacks targeted knowledge of protein–ligand interaction features necessary for structure-based drug design. To bridge the gap between unconditional molecule generation and structure-aware specific generation, we introduce a fine-tuning stage.

At this stage, we keep the ESM2 (650M) model (Lin et al. 2022) frozen to extract semantically meaningful representations from the protein sequences, which are subsequently encoded into feature embeddings. These protein embeddings are then incorporated into the model’s embedding layer through a cross-attention mechanism, enabling the network to effectively capture contextual interactions between the protein environment and the molecular representations. Finally, the combined embedding layer is optimized under the autoregressive training framework as formulated in Equation (13).

#### 2.4.3. Reinforcement learning fine-tuning

Following self-supervised fine-tuning, a Policy Gradient method is employed to optimize the resulting policy $\pi_{\theta }$ (Ziegler et al. 2019), thereby enhancing generation diversity and steering the molecular generation process. This approach balances the exploration of chemical space with semantic consistency by minimizing the divergence from a reward-adjusted prior. The objective function is defined as:


(14) $$L(\theta )=E_{S∼\pi_{\theta }}\left[{\left(\text{log}\;\pi_{\text{prior}}(S)+\sigma R(S)-\text{log}\;\pi_{\theta }(S)\right)}^{2}\right]$$

where $\pi_{\text{prior}}$ serves as a frozen semantic anchor to prevent policy collapse, and σ regulates the trade-off between retaining prior knowledge and maximizing rewards.

*Reward function design.* The reward function R(S) integrates docking affinity, drug-likeness (QED), and synthetic accessibility (SA) using a weighted sum:


(15) $$\begin{array}{c} R(S)=w_{\text{dock}}\cdot f(S_{\text{dock}},b_{\text{dock}})+w_{\text{qed}}\cdot \\ f(\text{QED},b_{\text{qed}})+w_{\text{sa}}\cdot f(\text{SA},b_{\text{sa}}) \end{array}$$

Weights are set to $w_{\text{dock}}=0.4$, $w_{\text{qed}}=0.3$, and $w_{\text{sa}}=0.3$. Each metric is normalized via a sigmoid function $f(x,b)={\left[1+\text{exp}(-k(x-b))\right]}^{-1}$ with a scaling factor k=10. Empirical thresholds are set as $b_{\text{dock}}=-3.0$, $b_{\text{qed}}=0.2$, and $b_{\text{sa}}=0.5$.

## 3. Results

This section presents the results for unconditional and protein-aware molecule generation. Training configurations, hyperparameters, and extended studies for each task are detailed in the Supplementary Data.

### 3.1. Unconditional drug design

#### 3.1.1. Dataset

We acquired approximately 18 million raw molecular structures in SMILES format from Uni-Mol (Zhou et al. 2023). The functional groups of these molecules were annotated according to the extraction workflow detailed in Section 2.1.

The 18 million-molecule dataset was randomly split into training, validation, and test sets with a ratio of 0.9996:0.0002:0.0002. For performance evaluation, we specifically utilized the first 2,048 molecules from the test set (Vaswani et al. 2017).

#### 3.1.2. Baselines

To the best of our knowledge, no current molecule generation method explicitly integrates functional group numbers information to enable precise quantitative control. To evaluate this capability, we assess several general-purpose large language models—including GPT-4.1 (Fachada et al. 2025), DeepSeek-v3.1 (Liu et al. 2024), and Grok-4 (Adinath and Is 2025). As a baseline, we also train a Transformer model (Touvron et al. 2023) of comparable scale on the exact same dataset used for NumMolFormer. Additional training details are provided in Supplementary Data.

Since the transformer architecture cannot directly handle parallel sequences, we reconstructed the input sequences to preserve functional group numbers, as illustrated in Supplementary Data.

#### 3.1.3. Molecule quality evaluation

We adopt widely used metrics to evaluate the quality of generated molecules. Specifically, *Validity* measures the proportion of generated SMILES strings corresponding to chemically valid molecules. *Uniqueness* quantifies the fraction of distinct molecules, while *Novelty* indicates the proportion of molecules not present in the training set. *Lipinski* (Lipinski et al. 1997) reports the fraction of molecules satisfying all five of Lipinski’s rules for drug-likeness. Additionally, *QED* (Quantitative Estimate of Drug-likeness) (Bickerton et al. 2012) evaluates overall drug-likeness on a continuous scale, and *SA* (Synthetic Accessibility) (Ertl and Schuffenhauer 2009) measures the ease of chemical synthesis.

As shown in Table 1, NumMolFormer demonstrates superior performance in generating diverse and highly drug-like molecules. It achieves scores in Uniqueness and Novelty (1.000), indicating robust exploratory capabilities. Critically, our method outperforms both general large language models (e.g. GPT-4.1, DeepSeek-v3.1) and the task-specific trained Transformer in key pharmaceutical metrics, securing the highest scores in Lipinski compliance (0.938) and QED (0.644).

**Table 1:** The table compares existing large language models (GPT-4.1, Grok-4, DeepSeek-v3.1), Transformer retrained on our dataset, and NumMolFormer.

Metrics	**Large Language Models**			Trained	Our
	GPT-4.1	Grok-4	DeepSeek-v3.1	Transformer	
General properties					
Validity (↑)	0.612	0.080	**0.954**	0.347	0.738
Uniqueness (↑)	0.981	0.998	0.884	0.994	**1.000**
Novelty (↑)	0.980	0.998	0.985	0.998	**1.000**
Lipinski (↑)	0.514	0.750	0.812	0.923	**0.938**
QED (↑)	0.590	0.539	0.628	0.631	**0.644**
SA (↑)	0.794	0.747	**0.882**	0.763	0.800
Functional group match					
Mean Squared Error (↓)	0.553	0.412	0.519	0.290	**0.121**
Exact Match Rate (↑)	0.006	0.000	0.005	0.106	**0.362**
Fuzzy Match Rate (↑)	0.217	0.187	0.208	0.361	**0.814**

Metrics with an upward arrow (↑) indicate higher values are better, while metrics with a downward arrow (↓) indicate lower values are better. For each metric, the best result is highlighted in **bold**.

#### 3.1.4. Functional group constraint evaluation

To quantitatively evaluate the model’s compliance with functional group constraints, we adopted three metrics. First, the Mean Squared Error (MSE) was computed between the target and generated molecules based on the distribution of 27 functional group numbers. Second, the Exact Match Rate (EMR) was defined as the proportion of molecules that fully satisfy all given constraints. Third, the Fuzzy Match Rate (FMR) measured the fraction of molecules whose functional group numbers fell within a deviation of ±3 from the targets.

As presented in Table 1, our model exhibits stronger controllability, successfully generating molecules under both strict and relaxed constraints, and significantly outperforms existing baselines. Furthermore, it is noteworthy that while DeepSeek-v3.1 achieves high pharmaceutical metrics, it exhibits poor adherence to functional group constraints, failing to generate molecules aligned with specified conditions.

### 3.2. Pocket-aware drug design

#### 3.2.1. Dataset

Following previous works (Peng et al. 2022, Guan et al. 2023b), we select 100 protein pockets from CrossDocked 2020 (Francoeur et al. 2020), leading to 100 000 pairs of pocket-ligand complexes for training, with 100 novel complexes as references for evaluation.

#### 3.2.2. Baselines

We compare NumMolFormer against various baselines for pocket-aware molecule generation, including AR (Luo et al. 2021), CVAE (Masuda et al. 2020), GraphBP (Liu et al. 2022a), Pocket2Mol (Peng et al. 2022), TargetDiff (Guan et al. 2023a), DecompDiff (Guan et al. 2023b), DrugGPS (Zhang and Liu 2023), and FLAG (Zhang et al. 2023).

#### 3.2.3. Setup

In alignment with previous works (Peng et al. 2022), we evaluated 100 molecules generated for each protein pocket, resulting in a total of 10 000 complex pairs for analysis.

We evaluate the performance across multiple dimensions using several metrics, including *Vina Score* (binding affinity), *QED* (drug-likeness), *SA* (Synthetic Accessibility), *Success Rate* [the proportion of molecules satisfying Vina Dock<−8.18, QED>0.25, and SA>0.59 (Guan et al. 2023b)], *Lip* (Lipinski’s Rule of Five), and *Div* (Diversity) computed as the average pairwise Tanimoto distances.

#### 3.2.4. Generated molecule evaluation

**Figure 2:**
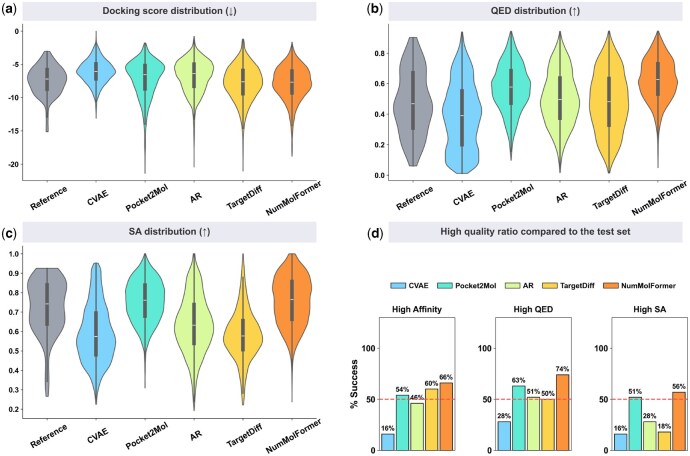
NumMolFormer achieves the state-of-the-art performance compared to baselines. (a–c) Distributional analysis of physicochemical properties, including Docking Score, QED, and SA. (d) Success rates in generating high-quality candidates across three criteria.

As shown in Table 2 and Fig. 2, molecules generated by NumMolFormer outperform baseline models on most metrics, particularly in QED, SA, and Success Rate. Remarkably, NumMolFormer even exceeds the dataset reference values across all metrics.

**Table 2:** Performance of NumMolFormer and baselines on pocket-aware drug design.

Methods	**Vina Score (** ↓ **)**	**QED (** ↑ **)**	**SA (** ↑ **)**	**Success Rate (** ↑ **)**	**Lip (** ↑ **)**	**Div (** ↑ **)**
Reference	−7.45	0.48	0.73	25.0%	4.25	–
AR	−6.75	0.51	0.63	7.1%	4.79	0.70
CVAE	−6.11	0.39	0.59	4.1%	4.03	0.65
GraphBP	−4.80	0.43	0.49	0.1%	4.45	0.79
Pocket2Mol	−7.15	0.56	0.74	24.4%	4.89	0.69
TargetDiff	−7.80	0.48	0.58	10.5%	4.49	0.72
DecompDiff	**−8.39**	0.45	0.61	24.5%	4.40	0.68
DrugGPS	−7.27	0.62	0.74	–	4.92	0.68
FLAG	−6.96	0.55	0.74	–	4.87	0.70
NumMolFormer	−7.84	**0.65**	**0.76**	**38.35%**	**4.93**	**0.90**

(↑)/(↓) denotes that a higher/lower value is better. The best result in each column is **bolded**.

The substantial boost in success rate demonstrates that incorporating functional-group triples—specifically quantitative data—effectively balances binding affinity with physicochemical properties, enabling the design of potent molecules with superior drug-likeness and synthesizability.

#### 3.2.5. Functional group analysis

**Figure 3:**
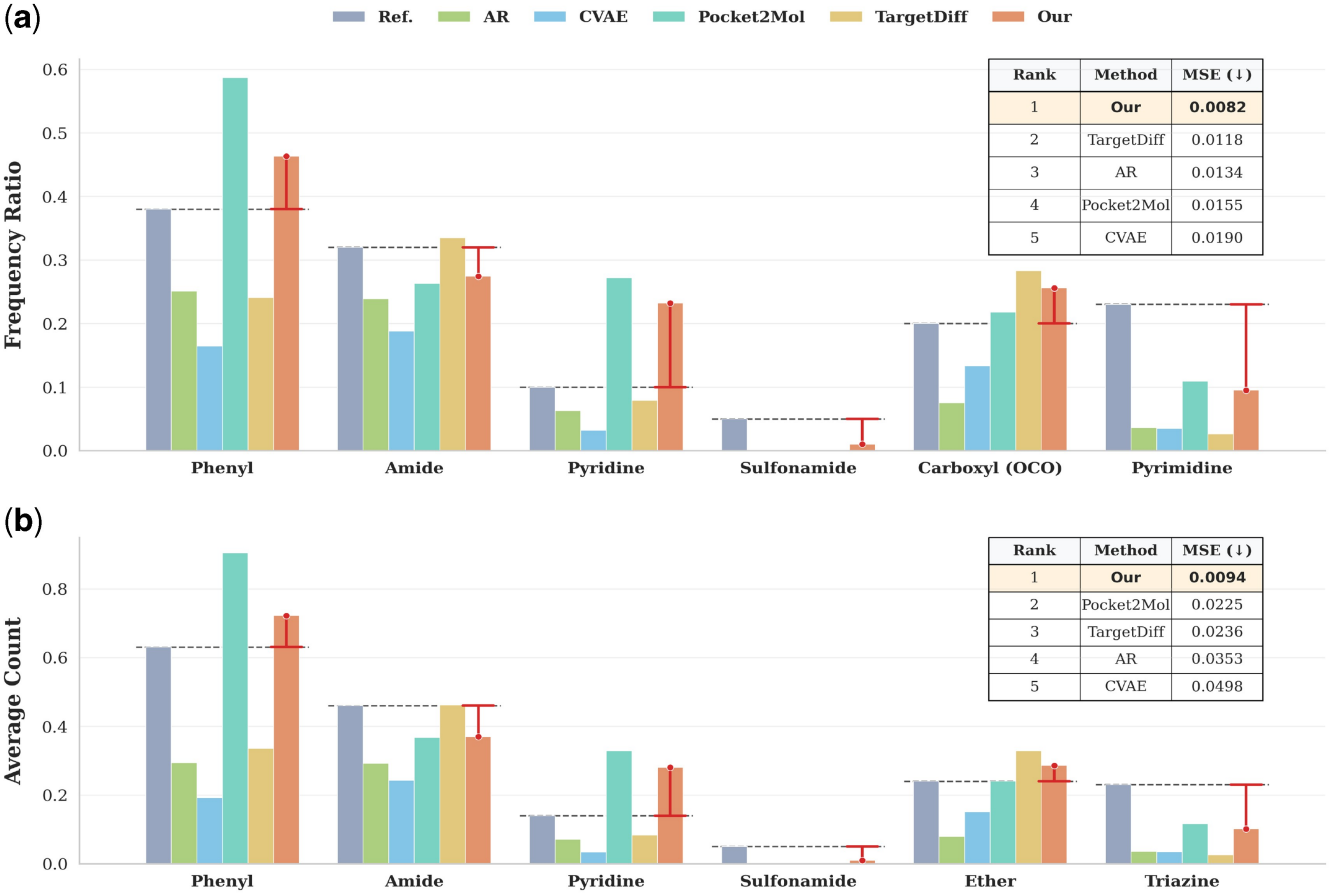
Structural fidelity and quantitative analysis of functional groups. The panels display the Frequency Ratio (top) and Average Number (bottom) across six representative functional groups. Compared to the training set (Ref.) and baselines, our method (Our) exhibits the optimal distributional fit. These results indicate that the model successfully decouples and learns underlying chemical patterns, generating chemically valid molecules across a wide range of group frequencies. Notably, in terms of average numbers, our method closely mirrors the reference data; this superior alignment validates the effectiveness of our architecture and its capability for precise quantitative control over molecular structures.

*Distribution consistency.* As shown in Fig. 3a, our method demonstrates superior fidelity in capturing the distribution of the six primary functional groups. While baseline models like CVAE and TargetDiff exhibit significant distributional discrepancies—either underestimating common moieties (e.g. Phenyl) or failing to balance complex polar groups, our approach maintains the closest alignment with the reference, a superiority quantified by an MSE of **0.0082**, which marks a significant improvement over TargetDiff (0.0118) and AR (0.0134).

*Quantitative performance.* Additionally, the average number per molecule for each functional group (Fig. 3b) further indicates that our model accurately learns the numerical distribution of chemical fragments, achieving an MSE of **0.0094**, thereby reflecting a more realistic structural composition.

## 4. Conclusion

NumMolFormer represents the first approach to explicitly guide structure-based molecule generation by incorporating the number of functional groups. It introduces a novel architecture that addresses the inherent limitations of Transformer in handling numerical data, thereby enabling effective encoding of functional group type, position, and number. As a result, NumMolFormer promotes the discovery of molecules with substantially enhanced drug-likeness, synthetic accessibility, and bioactivity. Although currently restricted to a predefined set of functional groups and in need of further large-scale validation, this work lays an important foundation for more precise and controllable molecular design.

## Supplementary Material

btag164_Supplementary_Data

## Data Availability

The datasets are available at http://www.github.com/zengzhicun/nummolformer.

## References

[btag164-B1] Achiam J , Adler S, Agarwal S et al Gpt-4 technical report. arXiv preprint, arXiv:2303.08774, 2023, preprint: not peer reviewed.

[btag164-B2] Adinath D , Is S. Chatgpt-5 and grok-4: a comparative analysis of advancements in nlp. *Available at SSRN 5547358*, 2025.

[btag164-B3] Bickerton GR , Paolini GV, Besnard J et al Quantifying the chemical beauty of drugs. Nat Chem 2012;4:90–8. 22270643 10.1038/nchem.1243 PMC3524573

[btag164-B4] Corsello SM , Bittker JA, Liu Z et al The drug repurposing hub: a next-generation drug library and information resource. Nat Med 2017;23:405–8. 28388612 10.1038/nm.4306 PMC5568558

[btag164-B5] Dziri N , Lu X, Sclar M et al Faith and fate: limits of transformers on compositionality. Adv Neural Inf Process Syst 2023;36:70293–332.

[btag164-B6] Ertl P , Altmann E, McKenna JM. The most common functional groups in bioactive molecules and how their popularity has evolved over time. J Med Chem 2020;63:8408–18. 32663408 10.1021/acs.jmedchem.0c00754

[btag164-B7] Ertl P , Schuffenhauer A. Estimation of synthetic accessibility score of drug-like molecules based on molecular complexity and fragment contributions. J Cheminform 2009;1:8. 20298526 10.1186/1758-2946-1-8 PMC3225829

[btag164-B8] Fachada N , Fernandes D, Fernandes CM et al Gpt-4.1 sets the standard in automated experiment design using novel python libraries. Future Internet 2025;17:412.

[btag164-B9] Ferreira LG , Dos Santos RN, Oliva G et al Molecular docking and structure-based drug design strategies. Molecules 2015;20:13384–421. 26205061 10.3390/molecules200713384 PMC6332083

[btag164-B10] Francoeur PG , Masuda T, Sunseri J et al Three-dimensional convolutional neural networks and a cross-docked data set for structure-based drug design. J Chem Inf Model 2020;60:4200–15. 32865404 10.1021/acs.jcim.0c00411 PMC8902699

[btag164-B11] Guan J , Qian WW, Peng X et al 3d equivariant diffusion for target-aware molecule generation and affinity prediction. In *The Eleventh International Conferenceon Learning Representations*, 2023a.

[btag164-B12] Guan J , Zhou X, Yang Y et al Decompdiff: diffusion models with decomposed priors for structure-based drug design. In *International Conference on Machine Learning*. PMLR, 2023b, 11827–11846.

[btag164-B13] Hann MM , Leach AR, Harper G. Molecular complexity and its impact on the probability of finding leads for drug discovery. J Chem Inf Comput Sci 2001;41:856–64. 11410068 10.1021/ci000403i

[btag164-B14] Irwin JJ , Shoichet BK. Zinc- a free database of commercially available compounds for virtual screening. J Chem Inf Model 2005;45:177–82. 15667143 10.1021/ci049714 PMC1360656

[btag164-B15] Kong D , Huang Y, Xie J et al Molecule design by latent prompt transformer. Adv Neural Inf Process Syst 2024;37:89069–97.

[btag164-B16] Landrum G et al Rdkit: a software suite for cheminformatics, computational chemistry, and predictive modeling. Greg Landrum 2013;8:5281.

[btag164-B17] Li Q. Application of fragment-based drug discovery to versatile targets. Front Mol Biosci 2020;7:180. 32850968 10.3389/fmolb.2020.00180 PMC7419598

[btag164-B18] Lin JH , Lu AY. Role of pharmacokinetics and metabolism in drug discovery and development. Pharmacol Rev 1997;49:403–49. 9443165

[btag164-B19] Lin Z , Akin H, Rao R et al Language models of protein sequences at the scale of evolution enable accurate structure prediction. bioRxiv 2022:500902, 2022, preprint: not peer reviewed.

[btag164-B20] Lipinski CA , Lombardo F, Dominy BW et al Experimental and computational approaches to estimate solubility and permeability in drug discovery and development settings. Adv Drug Deliv Rev 1997;23:3–25. 10.1016/s0169-409x(00)00129-0 11259830

[btag164-B21] Liu A , Feng B, Xue B et al Deepseek-v3 technical report. arXiv preprint, arXiv:2412.19437, 2024, preprint: not peer reviewed.

[btag164-B22] Liu M , Luo Y, Uchino K et al Generating 3d molecules for target protein binding. In *International Conference on Machine Learning (ICML)*, 2022a.

[btag164-B23] Liu S , Wang H, Liu W et al Pre-training molecular graph representation with 3d geometry. In *The 10th International Conference on Learning Representations (ICLR 2022)*. OpenReview. Net, 2022b.

[btag164-B24] Liu Z , Su M, Han L et al Forging the basis for developing protein–ligand interaction scoring functions. Acc Chem Res 2017;50:302–9. 28182403 10.1021/acs.accounts.6b00491

[btag164-B25] Luo S , Guan J, Ma J et al A 3d generative model for structure-based drug design. Adv Neural Inf Process Syst 2021;34:6229–39.

[btag164-B26] Mao F , Ni W, Xu X et al Chemical structure-related drug-like criteria of global approved drugs. Molecules 2016;21:75. 26771590 10.3390/molecules21010075 PMC6273477

[btag164-B27] Ragoza M , Masuda T, Koes DR. Generating 3d molecular structures conditional on a receptor binding site with deep generative models. Chem Sci 2022;13:2701–13. 10.1039/d1sc05976a PMC8890264 35356675

[btag164-B28] Peng X , Luo S, Guan J et al Pocket2mol: efficient molecular sampling based on 3d protein pockets. In *International conference on machine learning*. PMLR, 2022, 17644–17655.

[btag164-B29] Thawani A , Pujara J, Ilievski F et al Representing numbers in NLP: a survey and a vision. In *Proceedings of the 2021 conference of the North American chapter of the association for computational linguistics: human language technologies*, 2021, 644–656.

[btag164-B30] Touvron H , Lavril T, Izacard G et al Llama: open and efficient foundation language models. arXiv preprint, arXiv:2302.13971, 2023, preprint: not peer reviewed.

[btag164-B31] Van Montfort RL , Workman P. Structure-based drug design: aiming for a perfect fit. Essays Biochem 2017;61:431–7. 29118091 10.1042/EBC20170052 PMC5869280

[btag164-B32] Vaswani A , Shazeer N, Parmar N et al Attention is all you need. Adv Neural Inf Process Syst 2017;30:5998–6008.

[btag164-B33] Xie W , Zhang J, Xie Q et al Accelerating discovery of bioactive ligands with pharmacophore-informed generative models. Nat Commun 2025;16:2391. 40064886 10.1038/s41467-025-56349-0 PMC11894060

[btag164-B34] Zhang Z , Liu Q. Learning subpocket prototypes for generalizable structure-based drug design. In *International Conference on Machine Learning*. PMLR, 2023, 41382–41398.

[btag164-B35] Zhang Z , Min Y, Zheng S et al Molecule generation for target protein binding with structural motifs. In *The eleventh international conference on learning representations*. 2023.

[btag164-B36] Zhou G , Gao Z, Ding Q et al Uni-mol: a universal 3d molecular representation learning framework. 2023.

[btag164-B37] Zhu H , Zhou R, Cao D et al A pharmacophore-guided deep learning approach for bioactive molecular generation. Nat Commun 2023;14:6234. 37803000 10.1038/s41467-023-41454-9 PMC10558534

[btag164-B38] Ziegler DM , Stiennon N, Wu J et al Fine-tuning language models from human preferences. arXiv preprint, arXiv:1909.08593, 2019, preprint: not peer reviewed.

