# Supplementary material for: NumMolFormer: an explicit functional group number-guided framework for structure-based drug design
Source: Bioinformatics. 2026 Apr 7;42(4):btag164. doi: 10.1093/bioinformatics/btag164 (PMC13107960; doi:10.1093/bioinformatics/btag164)
Supplement: btag164_Supplementary_Data [file btag164_supplementary_data.pdf]

A. Related work

A.1. Advances in Structure-based Drug Design

Molecule design has been formulated as a conditional sequence generation task, as represented by methods such as **ResGen** (Zhang et al., 2023). Subsequent work has focused on better capturing the geometric and chemical features of binding pockets. For instance, **Pocket2Mol** (Peng et al., 2022) achieves atomic-resolution 3D generation by incorporating these structural constraints. To further improve physicochemical plausibility, explicit chemical priors have been introduced. **PGMG** (Zhu et al., 2023) employs pharmacophores as spatial and semantic prompts, while **TransPharmer** (Xie et al., 2025) integrates pharmacophore fingerprints with pre-trained models to efficiently generate novel bioactive structures. Despite these advances, explicit and precise control over the number of functional groups remains largely unexplored.

A.2. Numerical reasoning in Transformer

The standard Transformer architecture is inherently limited in handling numerical reasoning and arithmetic tasks. To enhance models' precise understanding and representation of numbers, several approaches have been proposed. For example, **LUNA** (Han et al., 2022) improves numerical comprehension by explicitly encoding relative positional information within numbers. **xVal** (Golkar et al., 2023) introduces a continuous numerical encoding scheme that extends embedding vectors with scalar values, replacing conventional discrete tokenization. Meanwhile, **(McLeish et al., 2024)** encode the position of each digit relative to the beginning of the number, leading to improved performance on arithmetic tasks.

B. Model details

As shown in Table 1, to determine the optimal architectural configuration, we performed a systematic hyperparameter search over the model depth (number of layers), width (embedding dimension), and the specialized magnitude parameter K.

We observe that scaling the embedding dimension from 256 to 768 and enhancing the model depth contributed the most substantial improvements, reducing the loss from 0.243 to below 0.20. This highlights the necessity of high-dimensional space for encoding complex chemical semantics. Interestingly, we identify an optimal balance in model depth; the 10-layer configuration outperforms the deeper 12-layer variant (0.171 vs 0.176), suggesting that the 10-layer structure provides sufficient representational power without incurring the optimization difficulties often associated with deeper networks. Furthermore, the magnitude parameter K proves critical; increasing K from 100 to 200 consistently improves convergence stability. Consequently, we selected the configuration highlighted in bold (10 layers, 12 heads, 768 embedding, $K = 200$) as the backbone for all subsequent experiments.

Table 1. Exploration of model hyperparameters.

Layer	Head	Embedding Dimension	K	Loss
12	12	768	200	0.176
**10**	**12**	**768**	**200**	**0.171**
10	12	768	100	0.192
8	12	768	200	0.198
8	8	768	100	0.216
8	8	256	100	0.243

C. Training details

C.1. Pretrain

Since the Transformer architecture cannot directly handle parallel sequences, we reconstructed the input sequences to preserve functional group numbers, as illustrated in Figure 1.

We trained the model on the full dataset for one epoch using four NVIDIA L20 GPUs, completing the process in less than 24 hours. The AdamW optimizer (Loshchilov and Hutter, 2017) was employed with a learning rate of 3×10^{-4} and a weight decay of 0.01. The batch size was set to 36, with gradient accumulation performed over 5 steps, resulting in an effective batch size of 720. A warmup phase of 0.25 was applied, followed by cosine learning rate decay. Notably, mixed-precision training with 16-bit floating point was not adopted, as it led to instability during training. This decision was driven by the fact that our model preserves the original numerical semantics, rendering it highly sensitive to numerical precision.

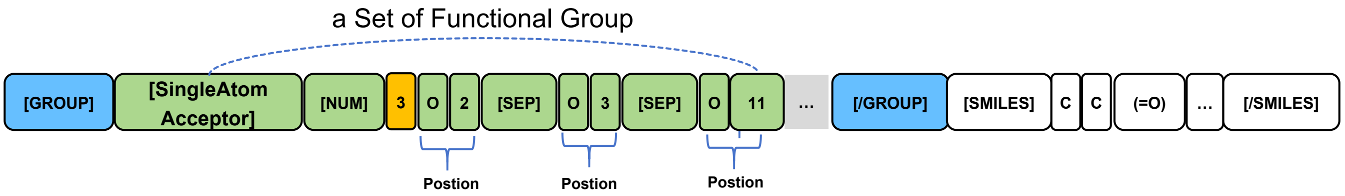

Figure 1 The sequences used for standard transformer pre-training.

C.2. Self-supervised training

Building on the pretraining setup, this stage continued to utilize the AdamW optimizer to ensure consistent parameter updates. However, to accommodate the protein-conditioned inputs and the reduced data scale relative to pretraining, we refined the hyperparameters: the learning rate was set to 2×10^{-4} and the warmup ratio was adjusted to 0.2. Additionally, gradient accumulation was disabled to increase update frequency and accelerate convergence on the current dataset.

C.3. Reinforcement learning finetuning

We performed 1,000 reinforcement learning (RL) steps for each protein pocket, utilizing a batch size of 32 and a learning rate of 1×10^{-4}. The parameter σ in Eq. 14 of the main manuscript was set to 100. Each RL process took less than 4 hours on a single NVIDIA L20 GPU with 128 CPU cores, while the computation of the Vina Score was carried out in parallel across the CPU cores.

D. Unconditional drug design

D.1. Numerical sensitivity evaluation for NumMolFormer

To quantitatively evaluate the numerical sensitivity of the model, we select seven representative sequences with the largest gradients from the test set. In each sequence, all functional group quantities are held constant except for *SingleAtomAcceptor*, whose number is varied from 1 to 7. For every sequence, we extract the final molecular embedding.

As shown in Figure 2, embedding similarity decreases progressively as the number of input functional groups rises. Moreover, the difference in functional group counts shows a strong negative correlation with embedding similarity, yielding a Pearson correlation coefficient of $\mathbf{-0.937}$. These findings suggest that a greater divergence in functional group quantities results in lower molecular embedding similarity, highlighting the model's sensitivity to variations in functional group compositions.

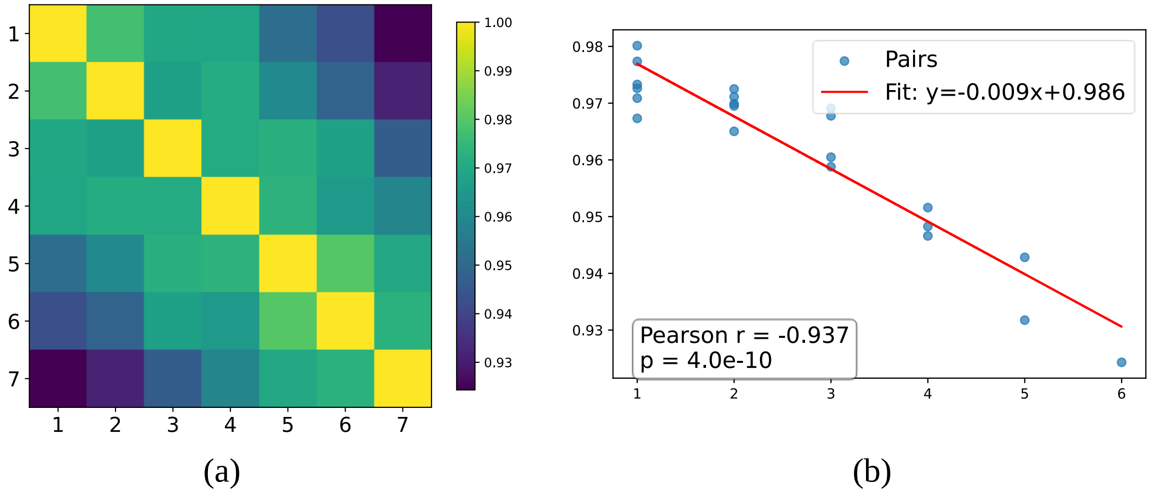

Figure 2 Numerical Sensitivity Analysis of NumMolFormer. (a) Cosine similarity of the embeddings from the model's last hidden layer for inputs with different numbers of functional groups. (b) Relationship between the differences in functional group numbers and the cosine similarity of their corresponding embeddings.

D.2. Numerical sensitivity evaluation for Transformer

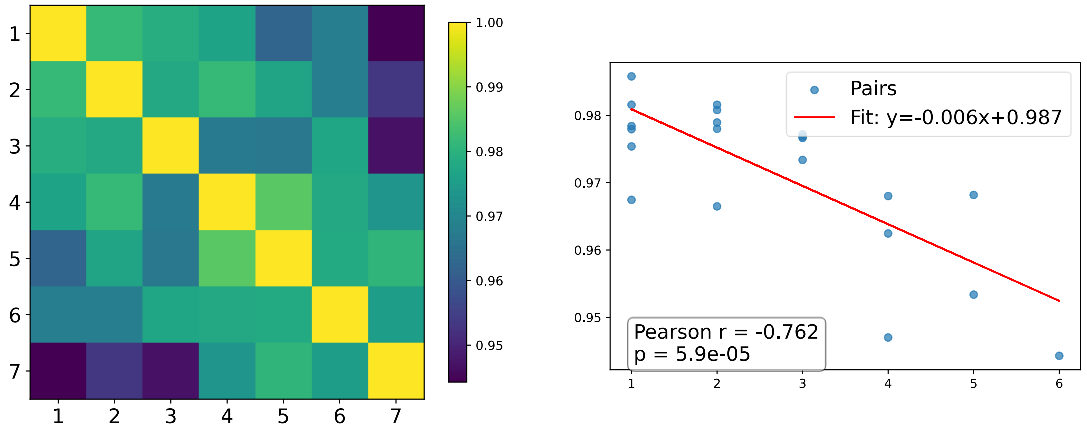

(a) (b)

Figure 3 Numerical Sensitivity Analysis of standard Transformer. (a) Cosine similarity of the embeddings from the model's last hidden layer for inputs with different numbers of functional groups. (b) Relationship between the differences in functional group numbers and the cosine similarity of their corresponding embeddings.

We further examine the capability of the standard Transformer model to capture numerical features after being pre-trained on a dataset augmented with quantitative functional group information. The experimental configuration follows that outlined in Section D.1.

Results show that although the standard Transformer demonstrates some ability to perceive numerical patterns, its precision remains limited. As seen in Fig. 3a, while the similarity matrix reveals a broad gradient trend, the progression is less smooth than in the enhanced model and exhibits noticeable nonlinear fluctuations. This finding is supported by Fig. 3b, which displays a moderate negative correlation ($r = -0.762$) but with considerable scatter in the data points. These outcomes indicate that the baseline model's embeddings reflect weaker consistency in mapping numerical differences to semantic similarities.

D.3. Ablation study

Table 2. Ablation studies comparing NumMolFormer with standard Transformer. (↑) / (↓) denotes that a higher / lower value is better. The best result in each column is **bolded**.

Methods	Validity (↑)	Uniqueness (↑)	Novelty (↑)	Lipinski (↑)	MSE (↓)
Transformer	0.426	1.000	0.997	0.927	0.283
Ours	**0.738**	**1.000**	**1.000**	**0.974**	**0.121**
Ours w/o Emb.	0.564	0.986	0.989	0.933	0.260
Ours w/o Attn.	0.628	0.981	0.952	0.927	0.240

We benchmarked NumMolFormer against the standard decoder-only transformer architecture and performed ablation studies to assess the effectiveness of individual components. Specifically, **w/o embed** excludes explicit numerical embedding, using only textual inputs; **w/o attention** replaces the dual-stream differential attention mechanism with standard attention applied to the numerically-augmented feature vectors.

As shown in Table 2, the numerical embedding module primarily enhances molecular validity and reduces the mean squared error (MSE), demonstrating its essential role in accurately encoding numerical features. The dual-stream differential attention module also positively affects both validity and MSE, underscoring its significance in capturing interactions among molecular representations. Removing both modules—i.e., reverting to a standard Transformer—results in the most substantial performance degradation, confirming that embedding and attention offer complementary advantages: embedding ensures the correctness of feature representation, while attention models dependencies between features.

D.4. Physicochemical property distribution

As shown in Figure 4, We assess the quality of molecules generated by the models by analyzing the distributions of QED and SA for the standard Transformer and NumMolFormer on the test set.

The results demonstrate that both architectures successfully learned the statistical patterns within the data, thereby effectively modeling the molecular feature space. However, subtle yet distinct differences were observed in their distribution trends: the outputs of the standard Transformer closely align with the boundaries of the ground truth values, whereas NumMolFormer exhibits a slight but consistent upward shift in both evaluated metrics. Given that higher QED scores and SA scores correspond to better drug-likeness and synthetic accessibility, respectively, this rightward shift suggests that NumMolFormer tends to generate molecular structures with more favorable physicochemical properties.

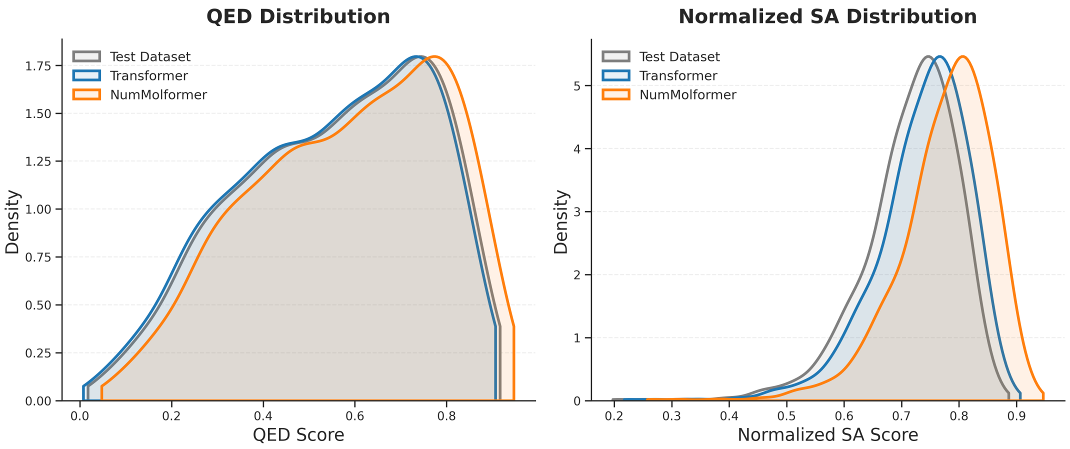

Figure 4 Comparative distributions of QED and Normalized SA. NumMolFormer (orange) demonstrates enhanced generative fidelity, closely tracking the ground-truth test distribution (grey) and outperforming the Transformer baseline (blue).

D.5. Detailed Evaluation of Functional Group Constraints

To rigorously evaluate the model's precision in functional group-guided generation, we conducted a sensitivity analysis using varying stringency thresholds for the Fuzzy Match Rate (FMR). Accordingly, we assessed the model's performance under increasingly stringent constraints: FMR (± 2) and FMR (± 1). These metrics measure the model's reliability in scenarios where even minor deviations in functional group counts could significantly impact the binding affinity or physicochemical properties of the generated molecules.

As shown in Table 3, our model consistently maintains a significant performance advantage, achieving an FMR (± 1) of $\mathbf{0.523}$. This stable retention of accuracy under increasingly strict constraints demonstrates that the framework effectively internalizes the precise mapping between numerical inputs and molecular structures.

Table 3. Sensitivity analysis of functional group-guided generation under varying stringency thresholds. The table compares baseline LLMs and a retrained Transformer against our model using the Fuzzy Match Rate (FMR) at different error tolerances $(\pm 3, \pm 2, \pm 1)$. Upward arrows (↑) indicate higher values are better, and the best results are highlighted in **bold**.

Metrics	Large Language Models			Trained	Our
	GPT-4.1	Grok-4	DeepSeek-v3.1	Transformer	
Fuzzy Match Rate(± 3) (↑)	0.217	0.187	0.208	0.361	**0.814**
Fuzzy Match Rate(± 2) (↑)	0.1069	0.1257	0.1014	0.189	**0.726**
Fuzzy Match Rate(± 1) (↑)	0.0359	0.0688	0.0285	0.132	**0.523**

E. Pocket-aware drug design

E.1. Model comparison

Pocket2Mol is an autoregressive model that generates molecules at the atomic level, following the classical atom-bond paradigm by sequentially adding atoms and forming bonds. **TargetDiff** is a diffusion-based method that generates molecular structures in 3D space, typically by predicting and refining atomic coordinates through a reverse diffusion process. Although both can capture global information, both model molecules solely at the level of atom types and bond types. **AR** proposes a 3D molecular generative model that directly generates candidate molecules with high binding affinity and good drug-likeness for given protein binding pockets. **CVAE** introduces a deep generative model-based approach for 3D molecular structure generation, which integrates an atom fitting algorithm to convert continuous densities into discrete molecular structures.

E.2. Structural realism and chemical feasibility

In addition to the docking score and physicochemical properties, the structural realism of generated molecules is a decisive factor for their practical utility. Current generative models often suffer from saturation bias, leading to an overabundance of excessively flexible, aliphatic-rich structures that miss the rigid scaffolds typical of drug molecules. We address this gap by investigating ring type distributions and conducting molecular saturation analysis.

E.2.1. Ring type distribution

Aromatic rings are fundamental to drug design, providing the rigid core required for $\pi - \pi$ stacking interactions and stable protein binding. Figure 5 compares the ratio of aromatic versus aliphatic rings across different models. As observed in the Reference dataset (ground truth), natural ligands typically exhibit a dominance of aromatic rings (Blue) over aliphatic ones (Orange).

However, baseline models including AR and TargetDiff significantly reverse this trend by generating an excessive number of aliphatic rings. This indicates their difficulty in mastering the principles of aromaticity and conjugation. In contrast, NumMolFormer successfully maintains the inherent preference for aromatic systems, showing a ring distribution profile in close agreement with the reference set. These results suggest that the explicit numerical processing mechanism employed in our approach enables the model to accurately capture the essential structure of drug-like molecules.

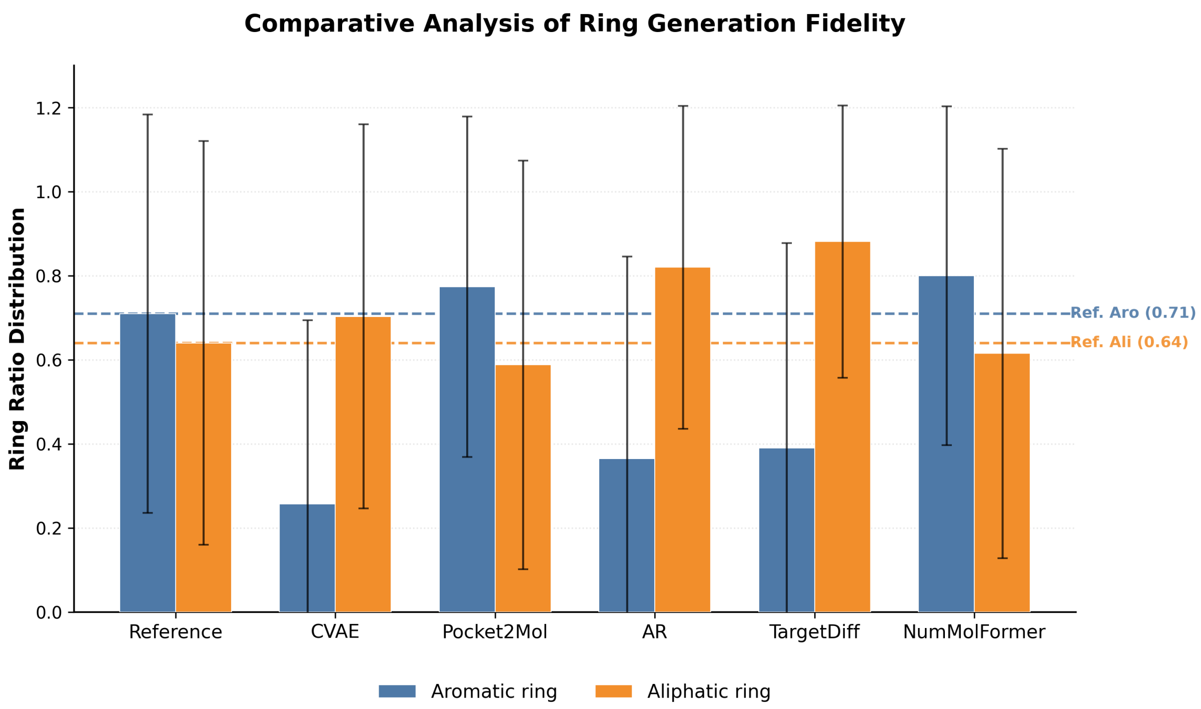

Figure 5 Ring aromaticity analysis. We assessed structural realism by comparing the proportion of aromatic (blue) rings to aliphatic (orange) rings. Baseline models (e.g., CVAE, AR, TargetDiff) show a clear bias toward aliphatic rings, indicating over-saturation, whereas NumMolFormer successfully preserves the dominance of aromatic rings characteristic of natural ligands (Reference).

E.2.2. Molecular saturation (F_{sp^3})

To further quantify structural complexity, we analyzed the fraction of sp^3-hybridized carbons (F_{sp^3}), a metric closely correlated with 3D complexity and solubility. While a certain degree of 3D character is desirable, excessively high F_{sp^3} often indicates "fluffiness"—molecules that are essentially blobs of saturated carbons lacking functional motifs.

As shown in Figure 6, baseline models such as CVAE and AR exhibit high F_{sp^3} scores (> 0.6), which aligns with the aliphatic preference observed in Figure 5. In contrast, NumMolFormer maintains a moderate F_{sp^3} level (approximately 0.35). Although this value is somewhat lower than the baseline, it allows the model to effectively avoid over-saturation. This suggests that NumMolFormer tends to generate compact and chemically dense structures, rather than spatially expanded yet chemically sparse molecules.

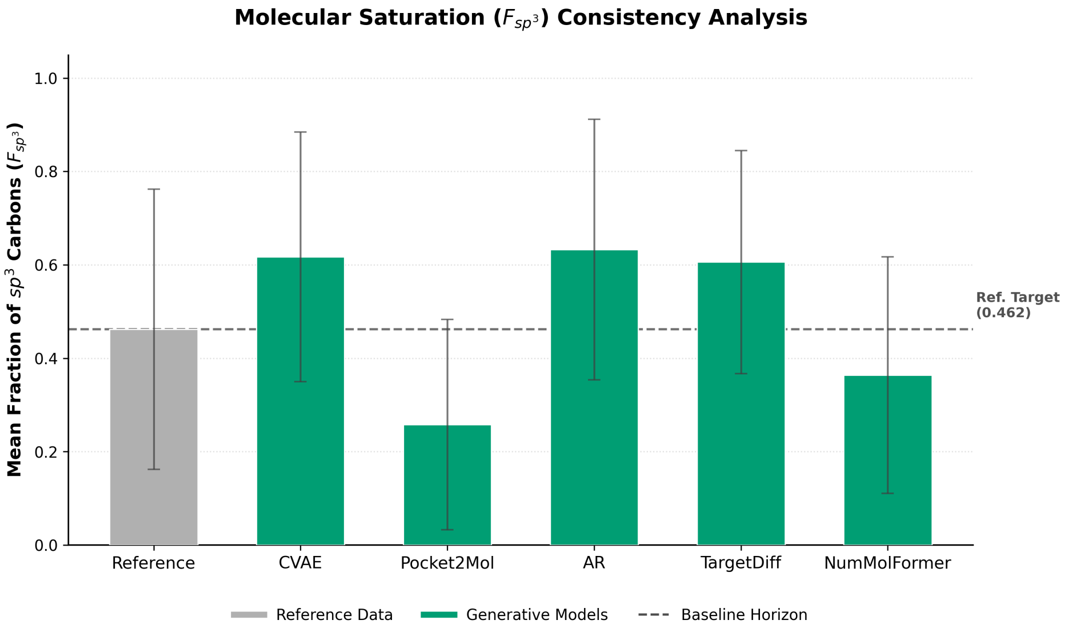

Figure 6 Comparison of molecular saturation (F_{sp^3}). In the baseline models, molecules generated by CVAE, AR, and TargetDiff exhibit relatively high F_{sp^3} values, indicating structurally oversaturated and predominantly aliphatic characteristics. In contrast, molecules produced by NumMolFormer demonstrate moderate F_{sp^3} values, which align more closely with the distribution observed in drug-like reference compounds, thereby avoiding excessive backbone flexibility.

E.3. Docking case

Table 4. Comparison of the reference molecule and molecules generated by our model for the pockets `ACE_HUMAN_650_1230_0` and `NEP_HUMAN_54_750_0`, including Vina Score, QED, and SA.

Molecule	SMILES	Vina Score (↓)	QED (↑)	SA (↑)
	ACE_HUMAN_650_1230_0			
Reference	C[C@H](NC(=O)O)C(=O)O	-3.6	0.483	0.820
Generated 1	Cc1coc(NCNc2cc(C)ccc2)=N1	**-7.2**	0.773	0.813
Generated 2	Cc1ncc(CCNc4cc(C)ncc4)o1	-6.1	**0.854**	**0.839**
Generated 3	Nc1coc(CCNc2cc(C)ncc2)=N1	-6.0	0.815	0.793
	NEP_HUMAN_54_750_0			
Reference	C[C@H](NC(=O)[C@H](Cc1ccc(-c2ccccc2)cc1)C[P@@](=O)(O)[C@H](C)N)C(=O)O	-5.7	0.463	0.731
Generated 1	CC(NC=C(Cc1ccc(-c2ccccc1)c(O)c2)C(=O)O)=O	**-8.9**	0.746	0.758
Generated 2	CC(NC(=O)C(Cc1ccc(-c2ccccc1)cc2)C(=O)O)=O	-6.9	0.848	0.745
Generated 3	CC(NC=C(Cc1ccc(-c2ccccc1)cc2)C(=O)O)=O	-6.4	**0.852**	**0.767**

In Table 4, we evaluate molecules generated by our model against reference ligands for the `ACE_HUMAN_650_1230_0` and `NEP_HUMAN_54_750_0` pockets. Assessment based on Vina Score, QED, and SA indicates that our model produces candidates with superior docking affinity while maintaining drug-likeness and synthetic ease. Corresponding docking poses are visualized in Figure 7.

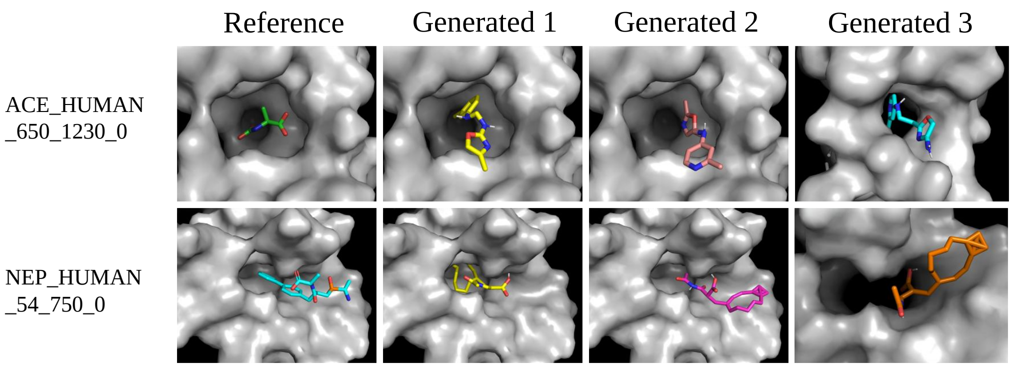

Figure 7 Docking results of the reference and generated molecules in `ACE_HUMAN_650_1230_0` and `NEP_HUMAN_54_750_0` pocket. Generated molecules outperform the reference compounds because they preserve the key functional groups and aromatic/ hydrophobic fragments necessary for protein binding, while generating structurally simpler scaffolds with high predicted affinity. The structural simplification enhances binding affinity, improves drug-likeness and synthetic accessibility.

F. Functional group

F.1. List of the functional groups

Table 5 lists the 27 functional groups extracted from RDKit.

Table 5. List of functional groups used in our study.

Category	Functional groups
Single-atom donors/acceptors	Single-atom hydrogen bond donors<br>Single-atom hydrogen bond acceptors
Acid-base groups	Acidic groups<br>Basic groups
Aromatic groups	Arom4<br>Arom5<br>Arom6<br>Arom7<br>Arom8
Zinc-binding groups	ZnBinder1<br>ZnBinder2<br>ZnBinder3<br>ZnBinder4<br>ZnBinder5<br>ZnBinder6
Branching groups	ThreeWayAttach<br>ChainTwoWayAttach
Common substituents	Nitro2<br>tButyl<br>iPropyl

F.2. Visualizations of 27 functional groups in molecules

Figure 8 presents visualizations of functional group structures, with each group highlighted by circles of different colors. The dataset does not contain the following four functional groups: ZnBinder2, ZnBinder3, ZnBinder5, and Arom8; therefore, they are not shown.

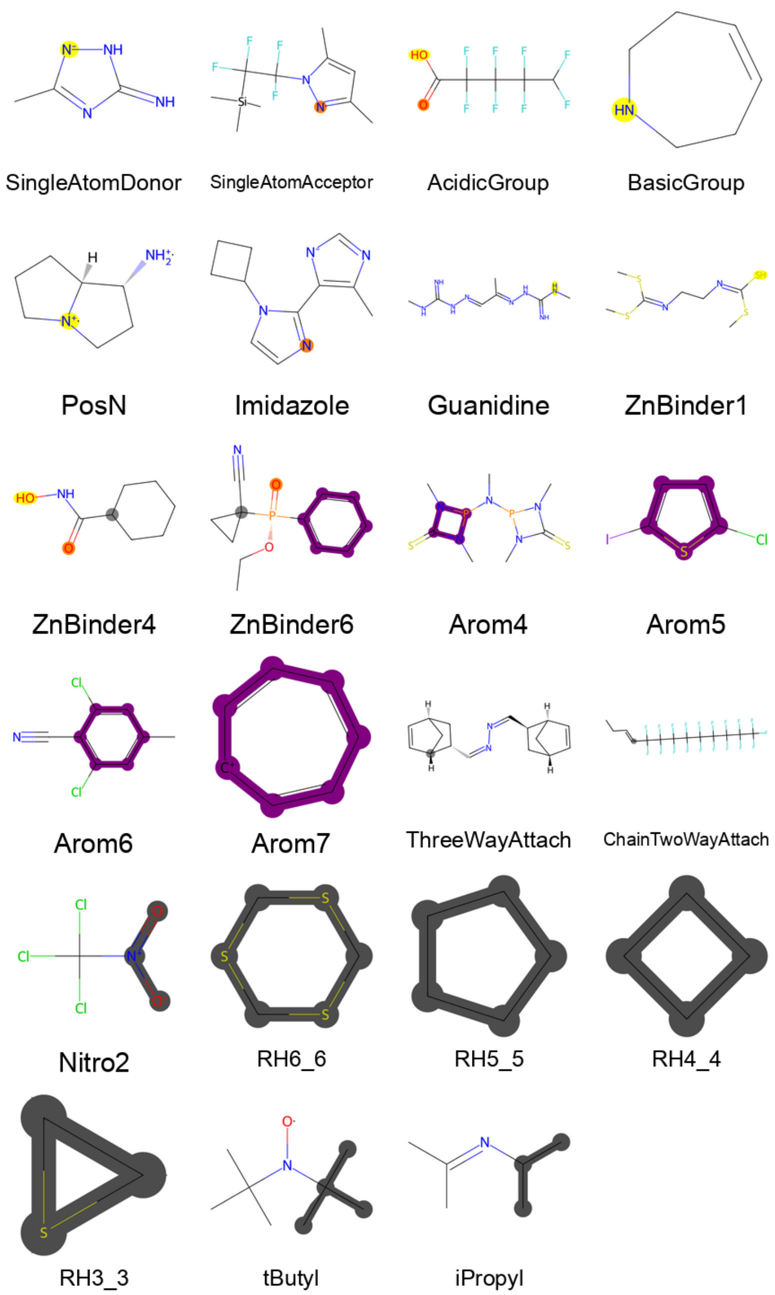

Figure 8 Detailed visualization of key pharmacophoric features and chemical substructures. The panel illustrates the discrete chemical motifs that contribute to ligand-protein interactions. Top rows showcase polar and charged groups crucial for electrostatic interactions (e.g., SingleAtomDonor/Acceptor, PosN). Middle rows depict aromatic and heterocyclic rings (Arom series, Imidazole) essential for $\pi - \pi$ stacking. Bottom rows display hydrophobic scaffolds and linkers (ThreeWayAttach, nitro groups, aliphatic chains). These identified features serve as the building blocks for the generated molecules, ensuring chemical validity and druggability.

References

S. Golkar, M. Pettee, M. Eickenberg, A. Bietti, M. Cranmer, G. Krawezik, F. Lanusse, M. McCabe, R. Ohana, L. Parker, et al. xval: A continuous number encoding for large language models. *arXiv preprint arXiv:2310.02989*, 2023.

H. Han, J. Xu, M. Zhou, Y. Shao, S. Han, and D. Zhang. Luna: language understanding with number augmentations on transformers via number plugins and pre-training. *arXiv preprint arXiv:2212.02691*, 2022.

I. Loshchilov and F. Hutter. Decoupled weight decay regularization. *arXiv preprint arXiv:1711.05101*, 2017.

S. McLeish, A. Bansal, A. Stein, N. Jain, J. Kirchenbauer, B. Bartoldson, B. Kailkhura, A. Bhatele, J. Geiping, A. Schwarzschild, et al. Transformers can do arithmetic with the right embeddings. *Advances in Neural Information Processing Systems*, 37: 108012–108041, 2024.

X. Peng, S. Luo, J. Guan, Q. Xie, J. Peng, and J. Ma. Pocket2mol: Efficient molecular sampling based on 3d protein pockets. In *International conference on machine learning*, pages 17644–17655. PMLR, 2022.

W. Xie, J. Zhang, Q. Xie, C. Gong, Y. Ren, J. Xie, Q. Sun, Y. Xu, L. Lai, and J. Pei. Accelerating discovery of bioactive ligands with pharmacophore-informed generative models. *Nature communications*, 16(1):2391, 2025.

O. Zhang, J. Zhang, J. Jin, X. Zhang, R. Hu, C. Shen, H. Cao, H. Du, Y. Kang, Y. Deng, et al. Resgen is a pocket-aware 3d molecular generation model based on parallel multiscale modelling. *Nature Machine Intelligence*, 5(9):1020–1030, 2023.

H. Zhu, R. Zhou, D. Cao, J. Tang, and M. Li. A pharmacophore-guided deep learning approach for bioactive molecular generation. *Nature Communications*, 14(1):6234, 2023.
